# Association of Gulf War Illness-Related Symptoms with Military Exposures among 1990–1991 Gulf War Veterans Evaluated at the War-Related Illness and Injury Study Center (WRIISC)

**DOI:** 10.3390/brainsci12030321

**Published:** 2022-02-27

**Authors:** Sarah T. Ahmed, Lea Steele, Peter Richardson, Shree Nadkarni, Sandhya Bandi, Mazhgan Rowneki, Kellie J. Sims, Jacqueline Vahey, Elizabeth J. Gifford, Stephen H. Boyle, Theresa H. Nguyen, Alice Nono Djotsa, Donna L. White, Elizabeth R. Hauser, Helena Chandler, Jose-Miguel Yamal, Drew A. Helmer

**Affiliations:** 1 Center for Health Services Research & Development Center for Innovations in Quality, Effectiveness, and Safety, Michael E. DeBakey VA Medical Center, Houston, TX 77030, USA; sarah.ahmed3@va.gov (S.T.A.); peter.richardson2@va.gov (P.R.); alice.nonodjotsa@va.gov (A.N.D.); donna.white2@va.gov (D.L.W.); 2 Section of Health Services Research, Department of Medicine, Baylor College of Medicine, Houston, TX 77030, USA; 3 Yudofsky Division of Neuropsychiatry, Department of Psychiatry and Behavioral Sciences, Baylor College of Medicine, Houston, TX 77030, USA; lea.steele@bcm.edu; 4 War Related Illness and Injury Study Center, Veteran Affairs (VA) New Jersey Healthcare System, East Orange, NJ 07018, USA; shree.nadkarni@va.gov (S.N.); sandhya.bandi@va.gov (S.B.); mazhgan.rowneki@va.gov (M.R.); helena.chandler@va.gov (H.C.); 5 Rutgers New Jersey Medical School, Newark, NJ 07103, USA; 6 Cooperative Studies Program Epidemiology Center-Durham, Durham VA Medical Center, Durham VA Health Care System, Durham, NC 27705, USA; kellie.sims@va.gov (K.J.S.); jacqueline.vahey@duke.edu (J.V.); elizabeth.gifford14@va.gov (E.J.G.); stephen.boyle@va.gov (S.H.B.); elizabeth.hauser@va.gov (E.R.H.); 7 Computational Biology and Bioinformatics Program, Duke University School of Medicine, Durham, NC 27705, USA; 8 Center for Child and Family Policy, Duke Margolis Center for Health Policy, Duke University Sanford School of Public Policy, Durham, NC 27708, USA; 9 Section of Gastroenterology and Hepatology, Department of Medicine, Baylor College of Medicine, Houston, TX 77030, USA; thn1@bcm.edu; 10 Department of Biostatistics and Bioinformatics, Duke University Medical Center, Duke Molecular Physiology Institute, Durham, NC 27701, USA; 11 Department of Biostatistics and Data Sciences, School of Public Health, University of Texas Health Science Center at Houston, Houston, TX 77030, USA; jose-miguel.yamal@uth.tmc.edu

**Keywords:** veteran, Gulf War illness, post-deployment health, War-Related Injury and Illness Study Center, pesticides, chemical alarms, Military-Ordered Protective Posture Level 4 (MOPP4) gear, pyridostigmine bromide

## Abstract

Veterans with difficult-to-diagnose conditions who receive care in the Department of Veterans Affairs (VA) healthcare system can be referred for evaluation at one of three specialty VA War-Related Illness and Injury Study Centers (WRIISC). Veterans of the 1990–1991 Gulf War have long experienced excess rates of chronic symptoms associated with the condition known as Gulf War Illness (GWI), with hundreds evaluated at the WRIISC. Here we provide the first report from a cohort of 608 Gulf War Veterans seen at the WRIISC who completed questionnaires on chronic symptoms (>6 months) consistent with GWI as well as prominent exposures during Gulf War deployment. These included veterans’ reports of hearing chemical alarms/donning Military-Ordered Protective Posture Level 4 (MOPP4) gear, pesticide use, and use of pyridostigmine bromide (PB) pills as prophylaxis against the effects of nerve agents. Overall, veterans in the cohort were highly symptomatic and reported a high degree of exposures. In multivariable models, these exposures were significantly associated with moderate-to-severe chronic symptoms in neurocognitive/mood, fatigue/sleep, and pain domains. Specifically, exposure to pesticides was associated with problems with concentration and memory, problems sleeping, unrefreshing sleep, and joint pain. Use of MOPP4 was associated with light sensitivity and unrefreshing sleep and use of PB was associated with depression. We also evaluated the association of exposures with symptom summary scores based on veterans’ severity of symptoms in four domains and overall. In multivariable modeling, the pain symptom severity score was significantly associated with pesticide use (Odds ratio (OR): 4.13, 95% confidence intervals (CI): 1.78–9.57) and taking PB pills (OR: 2.28, 95% CI: 1.02–5.09), and overall symptom severity was significantly associated with use of PB pills (OR: 2.41, 95% CI: 1.01–5.75). Conclusion: Decades after deployment, Gulf War veterans referred to a VA tertiary evaluation center report a high burden of chronic symptoms, many of which were associated with reported neurotoxicant exposures during the war.

## 1. Introduction

Approximately 30–44% of the nearly 700,000 U.S. military personnel who served in the 1990–1991 Gulf War developed a complex of chronic, poorly understood symptoms in the wake of their deployment. This condition is commonly known as Gulf War Illness (GWI) and is characterized by persistent fatigue, pain, neurocognitive, gastrointestinal, respiratory, and dermatological symptoms that are not explained by familiar medical or psychiatric diagnoses [1,2,3]. Studies have identified a range of neurological, immune, and other biological measures that significantly distinguish veterans with GWI from healthy veterans [1], although no diagnostic test has yet been established [4]. GWI is therefore identified primarily based on veterans’ symptoms, many of which have persisted for decades [5,6]. Over the years, multiple GWI case definitions have been proposed and used in different studies [7,8]. The two most widely used definitions, endorsed for research purposes by the Institute of Medicine (IOM 2014), are the Centers for Disease Control and Prevention (CDC) case definition for chronic multisymptom illness (CMI) [9] and the Kansas GWI case definition [10].

The Gulf War was relatively brief, ending with a decisive victory by the U.S.-led Coalition forces in February 1991 after just six weeks of air strikes and four days of ground combat. A long list of deployment factors has been suggested as causes or contributors to GWI that includes psychological factors as well as multiple exposures unique to Gulf War deployment. This includes the hundreds of oil well fires that burned in Kuwait throughout most of 1991, vaccines not previously used by the military, and depleted uranium munitions [7]. Now, more than 30 years after the war, studies of Gulf War veterans have consistently identified a limited number of deployment exposures as the most prominent risk factors for GWI [1,7]. These include widespread use of pyridostigmine bromide (PB) pills taken by troops as prophylaxis against adverse effects of nerve agents [1,11], as well as extensive use and overuse of multiple types of pesticides [1,12,13]. In addition, chemical alarms and alerts for suspected exposure to chemical weapons occurred regularly during the Gulf War. The best-documented chemical weapons’ incident affected approximately 100,000 troops potentially exposed to low doses of the nerve agents sarin and cyclosarin following demolition of Iraqi weapons near Khamisiyah, Iraq [14]. Chemical nerve agents, PB, and organophosphate and carbamate pesticides are all acetylcholinesterase inhibitors with similar modes of action that, depending on dose and delivery, have the potential to exert adverse effects on the brain and nervous system [7,15].

In response to widespread reports of unexplained health problems after the Gulf War, the U.S. Department of Veterans Affairs (VA) established the VA Gulf War Registry in 1994 to provide standardized examinations to Gulf War veterans with health concerns. In 2001, VA established the War-Related Illness and Injury Study Centers (WRIISC) to provide in-depth assessment of veterans with unexplained symptoms and difficult-to-diagnose conditions. This included a highly symptomatic cohort of 1990–1991 Gulf War veterans, whose persistent health problems were sufficiently serious and complex to require the in-depth, specialized evaluations provided at the WRIISC [16,17].

For this initial report, we consolidated WRIISC self-reported clinical intake data on veteran-reported symptoms and military exposures to determine if symptomatic illness in this unique cohort of Gulf War veterans is associated with the most prominent deployment-related experiences and exposures of concern. This included veterans’ use of PB pills, pesticide exposures, and possible exposure to chemical warfare agents as indicated by hearing chemical alarms and/or donning Mission-Oriented Protective Posture Level 4 (MOPP4) gear in response to suspected chemical threats during deployment. We report associations between symptom burden and these exposures of interest in this cohort of Gulf War veterans.

## 2. Materials and Methods

### 2.1. Setting and Participants

For the current study, we assembled data from a cohort of 608 veterans who served in the 1990–1991 Gulf War and were evaluated at one of three WRIISC sites between 2008 and 2020. In 2001, VA established two WRIISC sites at the East Orange, NJ, and Washington, DC, VA Medical Centers (VAMCs); a third WRIISC was added in 2008 at the Palo Alto, CA, VAMC. Veterans are referred by local VA providers for evaluation at the WRIISC sites after thorough primary and secondary evaluation at their home facility. Inclusion in the current study cohort was limited to veterans who deployed to the Gulf War theater for any period between August 1990 and November 1991 and completed clinical intake questionnaires. The retrospective medical record review research protocol and waiver of informed consent and Health Insurance Portability and Accountability Act (HIPAA) authorization were approved by institutional review boards at Baylor College of Medicine and the Michael E. DeBakey VA Medical Center.

### 2.2. Data Collection and Questionnaires

Veterans complete a series of standard and WRIISC-specific questionnaires as part of the intake process for WRIISC evaluation. Questionnaires collected demographic, military, and deployment information, as well as information to inform WRIISC clinicians about veterans’ medical histories, current health problems, and experiences and concerns about effects of military exposures [16]. Although intake packets were not designed specifically to determine GWI case status using existing criteria, veterans in the cohort completed the WRIISC Pain and Fatigue questionnaire module. This instrument, adapted from the CDC Chronic Fatigue Syndrome Symptom Inventory [18], queries veterans about the presence and severity of a number of chronic symptoms included in symptom domains defined by the Kansas GWI and CDC CMI criteria, based on symptoms that have persisted for six months or longer. Veterans in the current cohort also completed the WRIISC Military Environmental Exposures Questionnaire, adapted from the U.S. Army Public Health Command’s Deployment Air Respiratory Exposures (DARE) Questionnaire [19]. This instrument asks veterans if they have ever been exposed to a series of deployment and military-related exposures. Specific to the current study, this included questions about exposure to PB pills (also referred to as nerve agent pyridostigmine pretreatment or NAPP), pesticides/insecticides/flea collars, and chemical alarms/MOPP4.

### 2.3. Data Management and Analyses

#### 2.3.1. Symptom Assessment

We evaluated all queried symptoms for which information on severity and duration > 6 months was available for two or more symptoms within the symptom domains defined by Kansas GWI criteria. This allowed determination of the presence and severity of multiple chronic symptoms associated with four GWI domains: neurocognitive/mood, fatigue/sleep, pain, and gastrointestinal symptoms (Table 1). Skin and respiratory domains contained only one symptom each on the intake packet and therefore were not included. Veterans reported whether each symptom had been present for six months or more, and if the symptom was mild, moderate, or severe.

All veterans in the cohort reported multiple symptoms of varying severity. To evaluate the occurrence of more pronounced and persistent symptomatology, we defined our primary outcomes for individual symptoms as having either (1) moderate-severe symptoms lasting 6 months or longer or (2) no symptom, mild symptoms, or symptoms lasting < 6 months. The presence/absence of each moderate-severe chronic symptom was tabulated for each patient. We also evaluated symptom burden within each symptom domain and overall by generating ordinal summary scores for the presence/severity of chronic symptoms consistent with Kansas domain scoring, where 0 = no symptom, 1 = mild symptom, 2 = moderate symptom, and 3 = severe symptom. After summing the scores within each domain and overall, low vs. high symptom burden was defined as having a total score below vs. above the median (i.e., the value closest to the 50th percentile) for the cohort, for each symptom domain and for all symptoms combined (see Supplementary Material). The potential symptom domain score varied by domain (0–12 for neurocognitive/mood; 0–9 for fatigue/sleep; 0–6 for pain; 0–9 for GI; 0–36 for total symptom summary score). The median symptom summary score in each of the four symptom domains were as follows: a score of 7 in the fatigue/sleep problems symptom domain, score of 5 in the pain symptom domain, score of 7 in the neurocognitive/mood symptom domain, and score of 5 in the gastrointestinal symptom domain. The median total symptom summary score was 24. Distributions of symptom domain scores and total symptom summary scores are shown in Supplementary Figure S1.

#### 2.3.2. Exposure Variables

The WRIISC Military Environmental Exposures Questionnaire includes questions on whether a veteran was ever exposed to a variety of military exposures, with responses of “yes”, “no”, or “don’t know”. For the current study, we compared symptom outcomes among veterans who responded “yes” vs. “no” to the three primary exposure questions of interest, worded as follows: (1) “Chemical alarms/MOPP4” (question used as an indicator of possible exposure to chemical weapons); (2) “Insecticides, pesticides, flea collars”; and (3) “Chemical nerve agent antidotes (pyridostigmine bromide or NAPP)”. We coded the “don’t know”’ as missing, with the final variable being a binary variable.

#### 2.3.3. Statistical Analyses

Initial analyses compared the proportion of veterans who reported each exposure of interest in demographic and military subgroups using chi square tests. We also compared the proportion of exposed vs. not exposed veterans who developed individual chronic symptoms using chi-square tests. Prevalence odds ratios (OR) and 95% confidence intervals (C.I.) were calculated to determine the strength of association of each exposure of interest with individual symptoms, and with having high vs. low symptom summary scores. Logistic regression was used to evaluate the independent association of each exposure of interest with individual symptom outcomes, as well as high vs. low symptom domain and overall summary scores. Multivariable models adjusted for effects of covariates (age, sex, military branch) as well as potential confounding effects of concurrent exposures. All analyses excluded missing values.

All analyses were performed using SAS version 9.4 (SAS Institute, Inc., Cary, NC, USA) and Stata version 15 (StataCorp, College Station, TX, USA).

## 3. Results

The study cohort consisted of 608 Gulf War veterans seen at the WRIISC for evaluation of difficult to diagnose conditions. Demographic, military, deployment, and health characteristics of the sample are summarized in Table 2. As shown, the mean age of the veterans at the time of evaluation was 49 years (standard deviation (SD) 5.7), and more than 80% were white males. Compared to the overall population of veterans who served in the 1990–1991 Gulf War, the WRIISC cohort included a higher proportion of Army veterans (58% of WRIISC veterans vs. 50% overall) and a lower proportion of Navy veterans (12% WRIISC vs. 23% overall) [20].

Of note, some veterans did not know or did not report if they had experienced one or more deployment exposures. Overall, out of the total 608 veterans, 509 (84%) reported whether they heard chemical alarms/used MOPP4, 393 (65%) reported on pesticide exposure, and 385 (63%) reported if they used PB pills. Among veterans who responded to each exposure item, 439 of 509 (86%) affirmed hearing chemical alarms or use of MOPP4 gear, 303 of 393 (77%) affirmed exposure to pesticides, and 305 of 385 (79%) reported use of PB/NAPP pills.

As anticipated, veterans in the cohort were highly symptomatic (Table 2), with at least 74% reporting one or more symptoms in any of the four symptom domains, and at least 37% reporting one or more moderate-severe chronic symptom in any of the four symptom domains. The most commonly reported moderate-severe symptoms (present in > 90% of Veterans) included sleeping problems, unrefreshing sleep, and muscle discomfort or pains/aches.

All deployment exposures differed significantly by branch of service with the highest frequency of all exposures reported by Army veterans (Table 3). In addition, hearing chemical alarms/use of MOPP4 gear differed by race, ethnicity, and military component, with the highest frequencies reported by non-White (91%), Hispanic (95%), and veterans in the active component (89%). Finally, significantly more Hispanic (93%) and active component veterans (83%) reported use of PB/NAPP pills, compared to other veterans.

The number and proportion of exposed and unexposed veterans who experienced moderate-severe chronic symptoms at the time of their WRIISC evaluation are detailed in Supplementary Table S1. For all three exposure categories, higher proportions of exposed veterans reported moderate to severe symptoms for all but two symptoms across the four symptom domains. Elevated proportions observed among the exposed were statistically significant (*p* < 0.05) in bivariate analyses for 16 of 36 comparisons. Exposure-related differences in symptom reporting were most pronounced for pesticides/insecticides/flea collars and for use of PB/NAPP pills.

OR and 95% C.I. for these bivariate (unadjusted) associations are shown in Table 4. Overall, hearing chemical alarms/using MOPP4 gear was significantly associated with sensitivity to light (OR: 2.26, 95% C.I.: 1.32–3.88) and unrefreshing sleep (OR: 2.67, 95% C.I.: 1.18–6.06). Exposure to pesticides was significantly associated with substantial problems concentrating (OR: 1.80, 95% C.I.:1.04–3.10) and remembering (OR: 1.77, 95% C.I.: 1.05–3.00) and sensitivity to light (OR: 2.04, 95% C.I.: 1.25–3.34) in the neurocognitive/mood domain; nausea (OR: 1.92, 95% C.I.: 1.09–3.37) and diarrhea (OR: 1.73, 95% C.I.: 1.06–2.81) in the gastrointestinal domain; unrefreshing sleep (OR: 3.07, 95% C.I.: 1.41–6.67) and prolonged fatigue (OR: 2.04, 95% C.I.: 1.15–3.6) in the fatigue/sleep problems domain; and pain in the joints (OR: 2.16; 95% C.I.: 1.14–4.09) in the pain domain. Exposure to PB/NAPP pills was significantly associated with substantial problems concentrating (OR: 1.99, 95% C.I.: 1.11–3.56), sensitivity to light (OR: 1.95, 95% C.I.: 1.17–3.28), and depression (OR: 2.48, 95% C.I.: 1.48–4.16) in the neurocognitive domain, nausea (OR: 1.85, 95% C.I.: 1.06–3.22) and stomach or abdominal pain (OR: 2.21, 95% C.I.: 1.32–3.69) in the gastrointestinal domain, and prolonged fatigue (OR: 1.92, 95% C.I.: 1.04–3.55) in the fatigue/sleep problems domain.

Fewer significant associations were identified after adjusting for effects of covariates and potential confounding by concurrent exposures (Table 4). Hearing chemical alarms/use of MOPP4 gear was independently associated with two symptoms: sensitivity to light (OR: 3.05, 95% C.I.: 1.07–8.70) and unrefreshing sleep (OR: 3.85, 95% C.I.: 1.07–13.86). Pesticide exposure was associated with five symptoms in three domains, including substantial problems concentrating (OR: 2.29, 95% C.I.: 1.04–5.02) and remembering (OR: 2.18, 95% C.I.: 1.04–4.56), sleep problems (OR: 3.06, 95% C.I.: 1.19–7.89), unrefreshing sleep (OR: 3.13, 95% C.I.: 1.09–8.98), and joint pain (OR: 2.46, 95% C.I.: 1.04–5.82). Use of PB/NAPP pills was independently associated with only one chronic symptom, depression (OR: 2.68, 95% C.I.: 1.28–5.60). Overall, pesticide use was significantly associated with the largest number of symptoms.

As previously described, symptom summary scores were calculated to provide dichotomous values for high vs. low symptom severity within each domain, and high vs. low symptomatology overall, based on cohort median values. In unadjusted analyses (Table 4), hearing chemical alarms/use of MOPP4 gear was significantly associated with high symptom severity in two domains as well as total symptom severity, pesticide exposure was associated with high severity in three domains as well as total symptom severity, and use of PB/NAPP pills was associated with high severity in two domains as well as total symptom severity. After adjusting for covariates and concurrent exposures in multivariable models, both pesticide use and use of PB/NAPP pills were independently associated with high pain severity. In addition, use of PB/NAPP pills was independently associated with high total symptom severity (OR: 2.41, C.I.: 1.01–5.75) in multivariable models. However, veterans’ reports of hearing chemical alarms/use of MOPP4 were not significantly associated with any summary symptom measure in adjusted models.

## 4. Discussion

Among 608 Gulf War veterans referred to the Veterans Health Administration’s (VHA) WRIISC program between 2008 and 2020 for specialty evaluation of persistent unexplained symptoms, we found evidence of significant associations between veterans’ symptom burden and deployment-related exposures. The three exposures evaluated (hearing chemical alarms/use of MOPP4 gear as a proxy for possible nerve agent exposure, pesticides, and PB/NAPP pills) are potential neurotoxicants [7,15] and were significantly associated with multiple individual symptoms in the neurocognitive, fatigue, and pain domains, after controlling for effects of concurrent exposures and demographic and military covariates. In addition, use of pesticides and PB/NAPP pills were significantly associated with high pain domain summary scores, and use of PB/NAPP pills was associated with high total symptom summary scores.

Among veterans evaluated at the WRIISC, pesticide exposure was associated with the greatest number of individual chronic symptoms in multivariable analyses. Veterans exposed to pesticides were more than twice as likely to report chronic problems with concentrating (OR: 2.29, 95% C.I.: 1.04–5.02) and remembering (OR: 2.18, 95% C.I.: 1.04–4.56), sleep problems (OR: 3.06, 95% C.I.:1.19–7.89), unrefreshing sleep (OR: 3.13, 95% C.I.: 1.09–8.98), and joint pain (OR: 2.46, 95% C.I.: 1.04–5.82). In addition, chemical alarms/use of MOPP4 gear was significantly associated with sensitivity to light (OR: 3.05, 95% C.I.: 1.07–8.70) and unrefreshing sleep (OR: 3.85, 95% C.I.: 1.07–13.86), and use of PB/NAPP pills was significantly associated with depression in adjusted models (OR: 2.68, 95% C.I.: 1.28–5.60).

These strong associations between individual symptoms and exposures were generally predictive of exposure associations with high vs. low symptom domain scores in bivariate analyses, but not in adjusted models. Among the four dichotomized symptom domain scores, only the high vs. low pain summary score was significantly associated with exposure to pesticides (OR: 4.13, 95% C.I.: 1.78–9.57) and use of PB/NAPP pills (OR: 2.28, 95% C.I.: 1.02–5.09), and high vs. low total symptom severity was associated with use of PB/NAPP pills (OR: 2.41, 95% C.I.: 1.01–5.75). These may reflect the most significant associations with exposures when multiple symptom types are considered together, as in the domain and overall summary scores. However, it is also possible that the unique features of our sample, including the relatively low number of veterans with no or few symptoms in multiple domains and the lack of complete data for all exposures, imposed limitations when evaluating associations in fully adjusted models, due to reduced sample size and/or evaluation of veteran subgroups that differed from the full sample. It is also possible that, while the symptom domains appear clinically relevant, the putative causal pathways between the exposures and individual symptoms may not result in the symptom clusters as proposed.

Overall, our findings are consistent with previous studies of Gulf War veterans that have identified pesticides and use of PB/NAPP pills as the most prominent risk factors for GWI, after adjusting for other exposures in theater [1,7,21,22,23,24], as well as with individual types of symptoms and symptom domains [7,25,26,27]. An association between GWI and indicators of possible exposure to chemical weapons during the Gulf War has, overall, been less consistent [1,7]. Our use of veterans’ reports of hearing chemical alarms/MOPP4 gear as a proxy for possible sarin/cyclosarin exposure, while intuitive, might potentially over- or underestimate the true possibility of exposure to chemical warfare agents. That is, there are circumstances in which chemical alarms are believed to have sounded in the absence of actual chemical weapon exposures and, conversely, chemical weapons exposures are believed to have occurred in the absence of chemical alerts (pp. 134–146, [7]). In our sample, veteran reports of hearing chemical alarms/using MOPP4 gear were significantly associated with two symptoms, but not with symptom summary scores.

A leading theory of the etiology of GWI is based on the potential for neurotoxicant exposures, such as those represented by our exposure variables of interest, to precipitate a sustained neuroinflammatory response, perhaps in genetically vulnerable individuals, producing diverse, multisystem chronic symptoms [1,7,15,28]. Our findings are consistent with this theory and highlight the independent associations between each type of neurotoxicant exposure and specific symptoms in this sample with a high degree of concurrent exposures.

The Gulf War veterans referred to WRIISC reported a very high symptom burden, as reflected by individual symptom frequencies and by domain, and total symptom summary scores. This was because veterans are referred to the WRIISC tertiary evaluation centers by their own physicians after local clinical work up did not provide an explanation for their symptoms. Although we did not collect data on symptom onset for the current study, it is likely that, consistent with previous studies [5,22,27,29], veterans may have been suffering with these symptoms for many years. Identification of significant associations between symptoms and deployment-related exposures in this unique clinical cohort with very high symptom burden reinforces the possibility of a causal relationship between these neurotoxicant exposures and GWI.

Our study has several important strengths, beginning with the unique nature of the study cohort. Veterans in this sizable clinical cohort (*n* = 608) were referred to one of three WRIISC tertiary care centers by their VA providers from all areas of the United States for unexplained, difficult-to-diagnose conditions. Veterans deployed to the Persian Gulf War during 1990–1991, with data for the current study collected between 2008 and 2020, reflecting the current plight of Gulf War veterans suffering with unexplained chronic multisymptom illness. The symptoms analyzed considered both duration and severity, and the exposure items were derived from a generally accepted assessment instrument. Finally, Gulf War deployment status and military service characteristics were verified by the WRIISC team at the time of evaluation.

Our study also has limitations to consider in interpreting our findings. First, it is important to emphasize that the WRIISC cohort is not representative of the general population of U.S. Gulf War veterans. Veterans in the sample were more symptomatic than Gulf War veterans overall, and also potentially experienced a higher burden of deployment exposures. All were users of VA healthcare services and were referred to the WRIISC for specialized evaluation. Second, the purpose of the questionnaires in the WRIISC intake packet was for clinical assessment. Data used for the study were not collected for research purposes and did not include the full range of symptoms required to define GWI. Further, since the queried exposures occurred more than 20 years prior to completion of the intake packet, there is potential for inaccurate recollection and recall bias. A substantial number of veterans did not know or did not report individual exposures, yielding reduced sample sizes for some analyses, particularly those that included multiple exposures and symptom types.

## 5. Conclusions

Our findings of significant and independent associations between chronic symptoms and military exposure to pesticides and PB/NAPP pills among 1990–1991 Gulf War veterans in the WRIISC cohort are consistent with previous research on risk factors for GWI. Corroborating these associations in this highly symptomatic cohort, evaluated at a VA tertiary referral center, extends our confidence in the possibility of a causal association and contributes to our understanding of potential underlying mechanisms of GWI.

## Figures and Tables

**Table 1:** Symptom items included in the analyses, grouped by symptom domain.

**Neurocognitive/Mood Symptoms:** Substantial problems concentrating Substantial problems remembering Sensitivity to light Depression

**Fatigue/Sleep Symptoms:** Prolonged fatigue/feeling of illness lasting longer than a day after mild exercise Unrefreshing sleep Sleeping problems

**Pain Symptoms:** Muscle discomfort or pain/aches Pain in joints

**Gastrointestinal Symptoms:** Diarrhea Nausea Stomach or abdominal pain

**Table 2:** Demographic, military, and health characteristics of 1990–1991 Gulf War veterans evaluated in the War-Related Illness and Injury Study Centers (WRIISC) cohort.

Age, Mean (SD) Years	49 (5.69)	
	*n* (%)	
**Sex**		
Male	534 (88)	
Female	73 (12)	
**Race**		
White	485 (84)	
Non-White	92 (16)	
**Ethnicity**		
Non-Hispanic	474 (88)	
Hispanic	47 (9)	
Unknown	17 (3)	
**Military branch of service**		
Army	333 (58)	
Navy	69 (12)	
Air Force	64 (11)	
Marine Corps	102 (18)	
Coast Guard	2 (0.3)	
Other	4 (0.7)	
**Military component**		
Reserve	159 (28)	
Active	410 (72)	
**Military Exposures ^1^**		
Chemical alarms/MOPP4	439 (86)	
Exposure to pesticides, insecticides, flea collars	303 (77)	
Exposure to PB pills/NAPP	305 (79)	
**Current symptoms present 6 months or longer**	**Any** ***n*** **(%)**	**Moderate-Severe** ***n*** **(%)**
**Neurocognitive/Mood Symptoms** Problems concentrating Problems remembering Sensitivity to light Depression	526 (95) 534 (96) 371 (82) 475 (93)	440 (78) 435 (76) 282 (50) 376 (66)
**Fatigue/Sleep Symptoms** Sleeping problems Unrefreshing sleep Prolonged fatigue > 1 day after mild exercise	545 (97) 561 (98) 505 (93)	518 (91) 532 (93) 465 (82)
**Pain Symptoms** Muscle discomfort or pains/aches Pain in joints	557 (97) 548 (96)	522 (91) 502 (88)
**Gastrointestinal Symptoms** Nausea Diarrhea Stomach or abdominal pain	336 (74) 414 (79) 469 (89)	210 (37) 322 (56) 381 (67)

Abbreviations: SD = standard deviation; MOPP4 = Mission-Oriented Protective Posture Level 4; PB = pyridostigmine bromide; NAPP = nerve agent pyridostigmine pretreatment. ^1^ Exposure *n* (%) represents the number and proportion of veterans who reported each exposure out of the total number who provided valid responses and does not include missing values.

**Table 3:** Demographic and military characteristics of Gulf War veterans by exposure status.

	Chemical Alarms/MOPP 4 (*n* = 509)		Pesticides, Insecticides, Flea Collars (*n* = 393)		PB/NAPP Pills (*n* = 385)	
	Unexposed *n* = 70 (14%)	Exposed *n* = 439 (86%)	Unexposed *n* = 90 (23%)	Exposed *n* = 303 (77%)	Unexposed *n* = 80 (21%)	Exposed *n* = 305 (79%)
**Age, years, mean (SD)**						
	50.5 (7.6)	49.3 (5.3)	51.0 (7.1)	49.3 (5.3)	50.2 (6.5)	48.9 (4.8)
**Sex, *n* (%)**						
Female	7 (12)	53 (88)	9 (21)	34 (79)	8 (17)	38 (83)
Male	63 (14)	385 (86)	81 (23)	268 (77)	72 (21)	266 (79)
Race * *n* (%)						
White	61 (15)	353 (85)	73 (23)	240 (77)	69 (22)	250 (78)
Non-White	7 (9)	68 (91)	12 (19)	52 (81)	11 (23)	37 (77)
Ethnicity ** *n* (%)						
Non-Hispanic	61 (15)	339 (85)	65 (21)	244 (79)	70 (23)	241 (77)
Hispanic	2 (5)	35 (95)	10 (36)	18 (64)	2 (7)	25 (93)
Unknown	2 (12)	15 (88)	2 (14)	12 (86)	0 (0)	9 (100)
Military Branch of Service *** *n* (%)						
Army	19 (6)	274 (94)	41 (18)	191 (82)	42 (18)	195 (82)
Navy	22 (45)	27 (55)	17 (43)	23 (58)	10 (30)	23 (70)
Air Force	14 (26)	39 (74)	9 (24)	28 (76)	13 (38)	21 (62)
Marine Corps	9 (10)	81 (90)	18 (28)	46 (72)	7 (11)	58 (89)
Coast Guard	0 (0)	2 (100)	0 (0)	1 (100)	1 (100)	0 (0)
Other	1 (33)	2 (67)	2 (50)	2 (50)	2 (67)	1 (33)
Military Component ** *n* (%)						
Reserve	25 (18)	112 (82)	21 (20)	86 (80)	28 (27)	75 (73)
Active	39 (11)	310 (89)	64 (24)	203 (76)	45 (17)	222 (83)

Row percentages within each exposure reported. Abbreviations: SD = standard deviation; MOPP4 = Mission-Oriented Protective Posture Level 4; PB = pyridostigmine bromide; NAPP = nerve agent pyridostigmine pretreatment. * Proportion of exposed vs. unexposed varied significantly (*p* < 0.05) by race for chemical alarms/MOPP4; ** proportion of exposed vs. unexposed varied significantly (*p* < 0.05) by ethnicity and by component for chemical alarms/MOPP4 and PB/NAPP pills; *** proportion of exposed vs. unexposed veterans varied significantly (*p* < 0.05) across branches of service for all three exposure categories.

**Table 4:** Association of chronic symptoms with military exposures among Gulf War veterans evaluated at the War Related Illness and Injury Study Center (WRIISC).

	Chemical Alarms/MOPP 4		Insecticides, Pesticides, Flea Collars		Use of PB/NAPP Pills	
**Moderate-severe symptoms, 6 months or longer**	**OR (95% C.I.)** (unadjusted)	**OR (95% C.I.) ^a^** (adjusted)	**OR (95% C.I.)** (unadjusted)	**OR (95% C.I.) ^a^** (adjusted)	**OR (95% C.I.)** (unadjusted)	**OR (95% C.I.) ^a^** (adjusted)
**Neurocognitive/Mood Symptoms**						
Problems concentrating	1.50 (0.83–2.72)	1.18 (0.41–3.41)	1.80 (1.04–3.10) *	**2.29 (1.04–5.02)** *	1.99 (1.11–3.56) *	1.55 (0.65–3.70)
Problems remembering	1.18 (0.68–2.04)	1.34 (0.51–3.54)	1.77 (1.05–3.00) *	**2.18 (1.04–4.56)** *	1.45 (0.81–2.59)	1.14 (0.51–2.59)
Sensitivity to light	2.26 (1.32–3.88) *	**3.05 (1.07–8.70)** *	2.04 (1.25–3.34) *	1.54 (0.72–3.28)	1.95 (1.17–3.28) *	1.34 (0.61–2.95)
Depression	1.66 (0.98–2.81)	1.43 (0.57–3.55)	1.13 (0.69–1.86)	0.96 (0.43–1.97)	2.48 (1.48–4.16) *	**2.68 (1.28–5.60)** *
Neurocognitive/mood symptom domain score: high vs. low	2.23 (1.27–3.92) *	2.69 (0.92–7.84)	1.61(0.97–2.66)	1.33 (0.62–2.84)	2.16 (1.25–3.72) *	2.04 (0.91–4.58)
**Fatigue/Sleep Symptoms**						
Sleeping problems	1.77 (0.80–3.88)	2.40 (0.71–8.07)	2.04 (0.99–4.21)	**3.06 (1.19–7.89)** *	1.39 (0.59–3.25)	1.12 (0.37–3.39)
Unrefreshing sleep	2.67 (1.18–6.06) *	**3.85 (1.07–13.86)** *	3.07 (1.41–6.67) *	**3.13 (1.09–8.98)** *	1.80 (0.71–4.55)	1.16 (0.34–3.96)
Prolonged fatigue > 1 day after mild exercise	1.43 (0.76–2.68)	1.98 (0.71–5.46)	2.04 (1.15–3.59) *	2.02 (0.90–4.54)	1.92 (1.04–3.55) *	1.32 (0.54–3.21)
Fatigue symptom domain score: high vs. low	1.71 (1.01–2.92) *	2.50 (0.91–6.85)	1.79 (1.08–2.96) *	2.06 (0.97–4.38)	1.73 (1.02–2.92) *	1.25 (0.57–2.73)
**Pain Symptoms**						
Muscle discomfort or pains/aches	1.46 (0.61–3.46)	1.82 (0.52–6.33)	1.49 (0.70–3.16)	1.46 (0.55–3.84)	0.93 (0.39–2.23)	0.74 (0.23–2.31)
Pain in joints	1.62 (0.79–3.32)	1.31 (0.43–3.97)	2.16 (1.14–4.09) *	**2.46 (1.04–5.82)** *	1.97 (0.98–3.93)	2.28 (0.90–5.75)
Pain symptom domain score: high vs. low	0.91 (0.53–1.54)	1.00 (0.37–2.72)	3.01 (1.73–5.23) *	**4.13 (1.78–9.57)** *	1.63 (0.96–2.76)	**2.28 (1.02–5.09)** *
**Gastrointestinal Symptoms**						
Nausea	0.99 (0.58–1.70)	1.04 (0.37–2.91)	1.92 (1.09–3.37) *	1.44 (0.66–3.14)	1.85 (1.06–3.22) *	1.69 (0.74–3.88)
Diarrhea	1.39 (0.83–2.32)	1.20 (0.49–2.90)	1.73 (1.06–2.81) *	1.52 (0.76–3.03)	1.59 (0.96–2.64)	1.27 (0.61–2.61)
Stomach or abdominal pain	1.65 (0.97–2.80)	2.11 (0.85–5.20)	1.54 (0.93–2.55)	1.46 (0.71–3.00)	2.21 (1.32–3.69) *	1.66 (0.79–3.48)
Gastrointestinal symptom domain score: high vs. low	0.99 (0.57–1.70)	1.02 (0.40–2.60)	1.81 (1.09–3.00) *	1.88 (0.91–3.84)	1.65 (0.98–2.76)	1.17 (0.56–2.45)
**Total symptom summary score: high vs. low**	1.85 (1.02–3.33) *	2.35 (0.75–7.35)	1.90 (1.12–3.24) *	2.14 (0.96–4.76)	2.80 (1.56–5.03) *	**2.41 (1.01–5.75)** *

^a^ Multivariable models adjusted for age, sex, branch of service, and concurrent exposures (hearing chemical alarms, pesticide exposure, use of PB/NAPP pills). (*) denotes association is statistically significant (*p* < 0.05); significant (*p* < 0.05), adjusted associations are shown in **bold type**. Abbreviations: MOPP4 = Mission-Oriented Protective Posture Level 4; PB = pyridostigmine bromide; NAPP = nerve agent pyridostigmine pretreatment; OR = odds ratio; C.I. = confidence interval.

## Data Availability

Not applicable.

## Supplementary Materials

The following are available online at https://www.mdpi.com/article/10.3390/brainsci12030321/s1: Table S1. Proportion of Gulf War veterans reporting moderate to severe chronic symptoms, by exposure status; Figure S1. Distribution of symptom summary scores in Gulf War veterans.
