# Supplementary material for: Association of Gulf War Illness-Related Symptoms with Military Exposures among 1990–1991 Gulf War Veterans Evaluated at the War-Related Illness and Injury Study Center (WRIISC)
Source: Brain Sci. 2022 Feb 27;12(3):321. doi: 10.3390/brainsci12030321 (PMC8946608; doi:10.3390/brainsci12030321)
Supplement: Supplementary file 1 [file brainsci-12-00321-s001.zip › brainsci-1578581-supplementary.pdf]

Table S1: Proportion of Gulf War Veterans reporting moderate to severe chronic symptoms, by exposure status

	Chemical Alarms/ MOPP4 (n=509)		Pesticides/Insecticides/ Flea Collars (n=393)		PB pills/ NAPP (n=385)	
	Unexposed (n=70)	Exposed (n=439)	Unexposed (n=90)	Exposed (n=303)	Unexposed (n=80)	Exposed (n=305)
	n (%)	n (%)	n (%)	n (%)	n (%)	n (%)
Neurocognitive/Mood Symptoms						
Substantial problems concentrating	47 (72)	338 (80)	57 (69)	233 (80)*	52 (70)	240 (82)*
Substantial problems remembering	51 (75)	329 (77)	55 (65)	226 (77)*	56 (73)	233 (80)
Sensitivity to light	23 (34)	225 (54)*	33 (38)	160 (56)*	30 (39)	161 (56)*
Depression	38 (57)	288 (69)	54 (62)	188 (65)	36 (48)	204 (70)*
Fatigue/Sleep Symptoms						
Sleeping problems	58 (87)	388 (92)	71 (85)	268(92)	68(89)	273(92)
Unrefreshing sleep	59 (87)	404 (95)*	73 (85)	276(95)*	68(91)	280(95)
Prolonged fatigue or feeling of illness lasting longer than a day after mild exercise	53 (78)	349 (83)	63 (72)	241(84)*	59(76)	245(86)*
Pain Symptoms						
Muscle discomfort or pains/aches	59 (89)	394 (92)	75(87)	265 (91)	71(91)	266 (90)
Pain in joints such as elbows, knees and fingers without redness and swelling	56 (84)	381 (89)	68 (79)	262(89)*	63(82)	266 (90)
Gastrointestinal Symptoms						
Nausea	25 (38)	160 (38)	19 (22)	103(35)*	21 (27)	119 (41)*
Diarrhea	34 (50)	246 (58)	40 (47)	177(61)*	39 (51)	182 (62)
Stomach or abdominal pain	37 (56)	289 (68)	52 (61)	209(71)	40 (52)	208 (71*)

Abbreviations: MOPP4 = Mission Oriented Protective Posture Level 4; PB = pyridostigmine bromide; NAPP = nerve agent pyridostigmine pretreatment.

Notes: Column percentages reported. Values with asterisk (*) denote differences between unexposed and exposed groups for that symptom at $p < 0.05$.

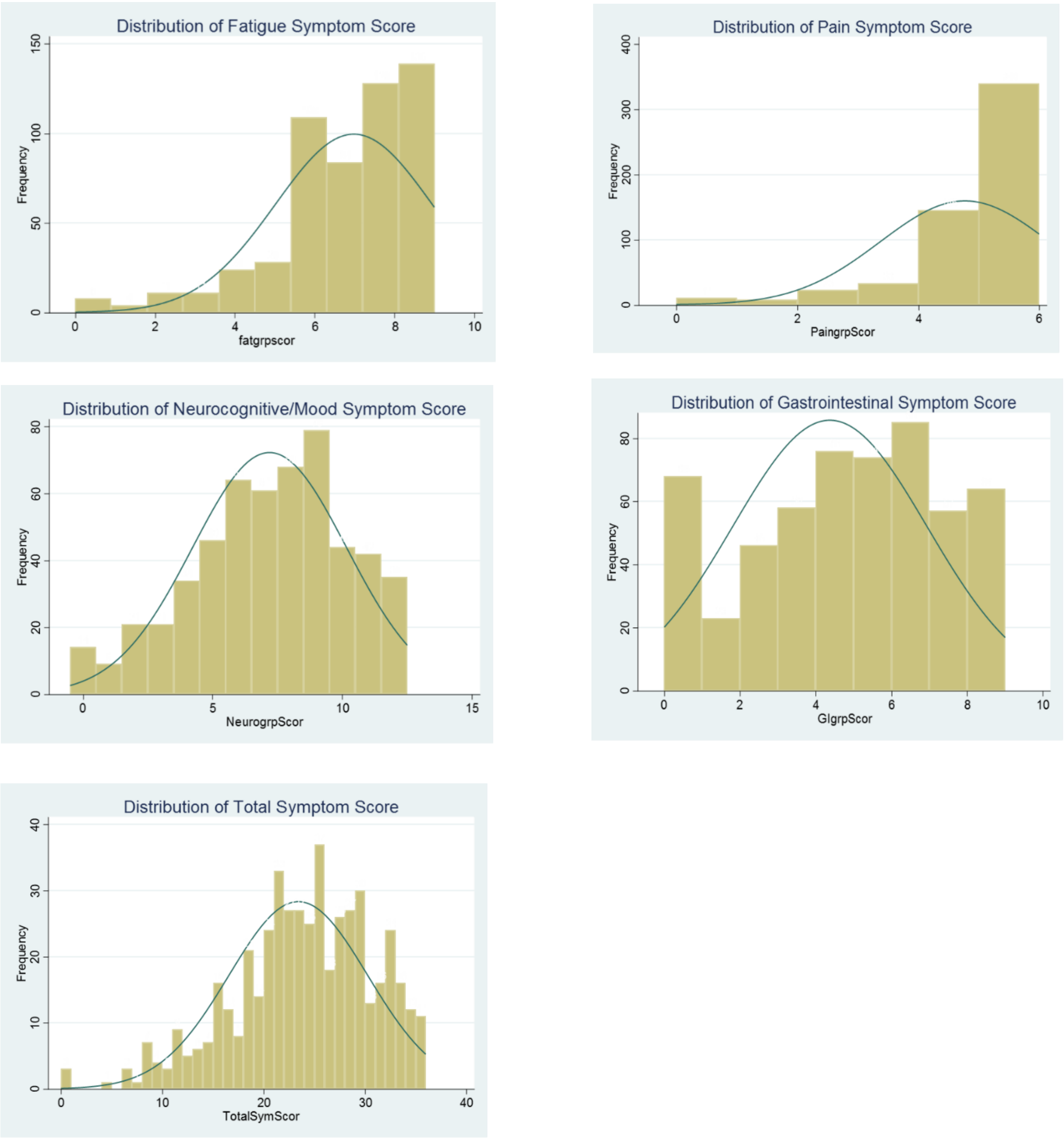

Supplementary Figure S1. Distribution of Symptom Summary Scores in Gulf War Veterans.
